# Managing corneal disease: focus on suppurative keratitis

**Published:** 2009-12

**Authors:** Madan P Upadhyay, Muthiah Srinivasan, John P Whitcher

**Affiliations:** President, BP Eye Foundation, Kathmandu, Nepal. Email: madanupadhyay@hotmail.com; Director and Chief of Cornea Services, Aravind Eye Hospital, Madurai, India.; Clinical Professor of Ophthalmology, Proctor Foundation, University of California, San Francisco, USA.

**Figure:**
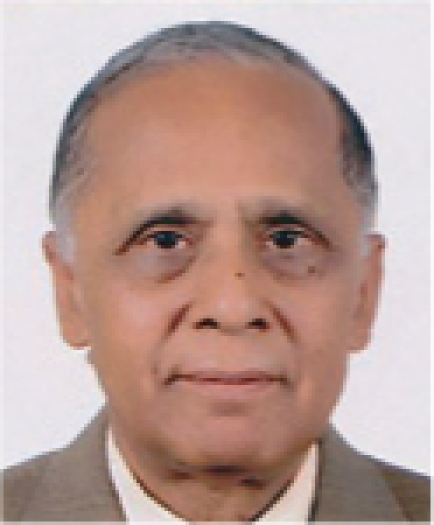


**Figure:**
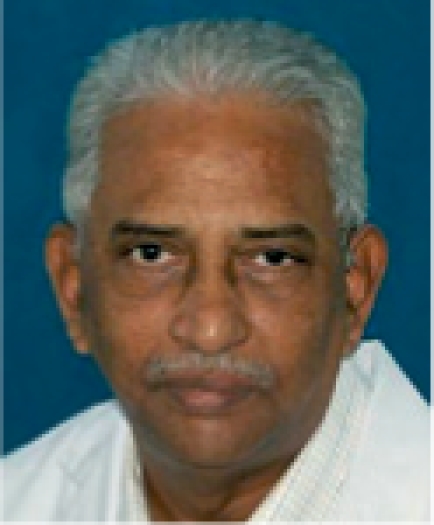


**Figure:**
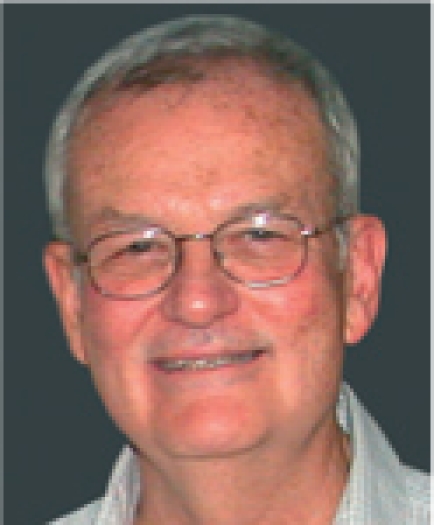


## Introduction

Infections of the cornea can lead to corneal opacity and blindness if not identified quickly and managed appropriately. The terms infective keratitis, suppurative keratitis, and microbial keratitis are all used to describe suppurative infections of the cornea. These are characterised by the presence of white or yellowish infiltrates in the corneal stroma, with or without an overlaying corneal epithelial defect, and associated with signs of inflammation (Figure 1).

**Figure:**
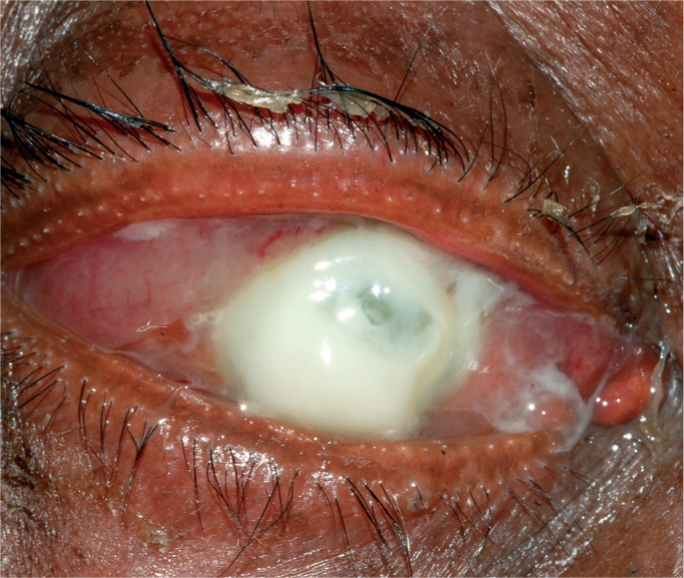
Figure 1. A severe bacterial ulcer caused by *Pseudomonas sp.* The gram negative (−ve) bacillus can cause complete destruction of the cornea within a few days. This cornea is at risk of perforation.

Dr M Srinivasan/Aravind Eye Hospital

**Figure:**
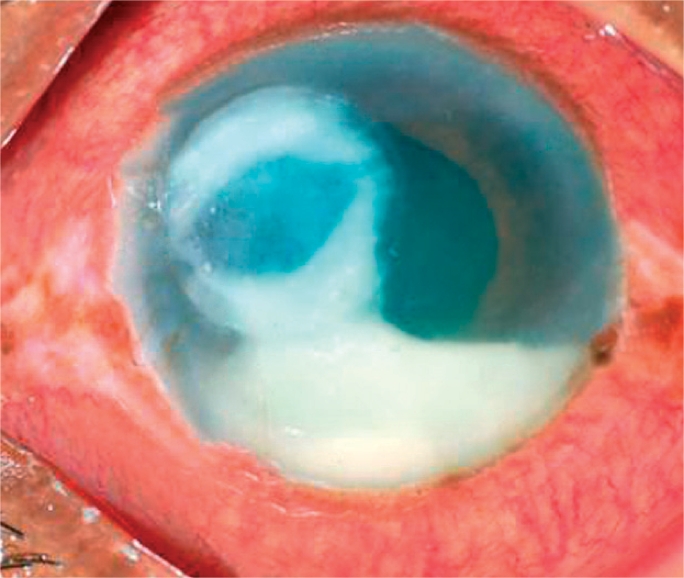
Figure 2. A bacterial ulcer. The eye is very red and inflamed. Note the ring infiltrate in the cornea and a large hypopyon in the anterior chamber.

Dr M Srinivasan/Aravind Eye Hospital

The common symptomatic complaints of patients with suppurative keratitis are as follows (all with varying degrees of severity):

- redness of the eye
- circum-corneal congestion (typically)
- pain
- blurring of vision
- photophobia
- watering of the eye.

The aim of this article is to review both bacterial and fungal keratitis, with an emphasis on identification and management at the primary, secondary, and tertiary levels. Guidelines for referral will be suggested.

## Fist steps in management

### History taking

History taking is an important step in management of corneal infection. If there has been an injury, ask when and where the injury was sustained, what the patient was doing at the time of injury, whether or not he or she sought help following the injury, and what treatment - including traditional eye medications - may have been used.

A past history of conjunctivitis may suggest that the infection is secondary to a conjunctival pathogen.

### Examination

#### 1. Visual acuity

Visual acuity should always be recorded in all cooperative patients. If it is not possible to record the visual acuity of a child, for example, a note of this should be made. Vision should be recorded first in the unaffected eye, then in the affected eye; with or without glasses. This provides a useful guide regarding the prognosis and response to treatment. It is also important documentation in the event of medico-legal issues.

#### 2. Examination of the cornea

A torch with a good source of focused light and a loupe for magnification are essential. A slit lamp microscope, if available, is always helpful, but not absolutely essential.

Another essential tool is fluorescein dye, either in a sterile strip or a sterile solution. Fluorescein stains any part of the cornea that has lost the epithelium, even due to a trivial injury, and appears brilliant green when viewed under blue light (Figure 3).

**Figure:**
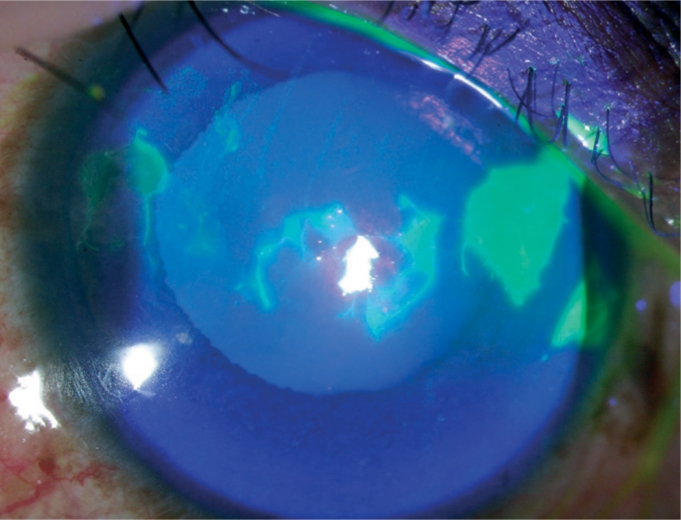
Figure 3. Fluorescein staining

#### 3. Corneal scrape

Diagnosis should be confirmed by obtaining a corneal scraping from the corneal lesion and subjecting it to laboratory testing at secondary or tertiary eye care facilities. See article on page 42.

## Management at primary level

A suppurative corneal ulcer is an ophthalmic emergency which should be referred to the nearest eye centre for proper management. The following are useful guidelines when referring the patient to the secondary eye care centre.

- **Do** apply antibiotic drops or ointment
- **Do** instruct patients and/or their accompanying persons to apply drops frequently until patients arrive at the centre
- **Do** instruct patients and/or their accompanying persons to avoid traditional medicines.
- **Do not** give systemic antibiotics; they are not helpful
- **Do not** use steroid drops and/or ointment; they can be dangerous
- **Do not** routinely patch the eye; it is not necessary.

## Management at secondary level

More complete management of corneal infections begins at the secondary level of eye care where there is an ophthalmologist and/or an ophthalmic assistant, or a physician trained in managing common eye diseases. At the secondary level:

- A corneal scraping should be taken (see page 42).
- The patient should be admitted to the hospital to ensure adequate treatment and frequent follow-up.

### Specific initial treatment

#### No fungal elements seen

- instil cefazolin 5% and gentamicin 1.4% drops hourly.
- Ciprofloxacin or ofloxacin is a good substitute for gentamicin and cephazolin. If it is not possible to administer hourly drops, a subconjunctival injection can be given.

#### Fungal elements seen

- Natamycin 5% drops hourly or freshly reconstituted amphotericin-B 0.15% as drops hourly. Antibiotics may have a limited role to play in such cases and may occasionally be harmful. Clinical judgment correlated with laboratory tests constitute the best guide in such cases.

### Adjunctive treatment

- Atropine 1% or homatropine 2% could be used twice a day to dilate the pupil; this helps to prevent synechiae and relieve pain
- Oral analgesics will help to minimise pain
- Anti-glaucoma medication may be advisable if the intraocular pressure is high
- Vitamin A supplementation may be helpful, particularly in countries where Vitamin A deficiency is markedly prevalent.

**Five A's** are a useful acronym to remember: **A**ntibiotic/antifungal, **A**tropine, **A**nalgesics, **A**nti-glaucoma medications, and Vitamin **A**.

### Subsequent management

Suppurative keratitis patients should be hospitalised and examined daily, if possible with a slit lamp, so that their response to treatment can be evaluated and the frequency of antibiotics adjusted accordingly.

Reduce the frequency of antibiotic administration when the patient experiences symptomatic improvement (less tearing and photophobia, relief from pain, and improvement in vision) and when the ulcer shows signs of improvement:

- decrease in lid oedema
- decrease in conjunctival chemosis and bulbar conjunctival injection
- reduction in density of the infiltrate and area of epithelial ulceration
- haziness of the perimeter of the ulcer and of the stromal infiltrate
- decrease in inflammation; cells, fibrin, and level of hypopyon
- dilatation of pupil.

In the case of bacterial infection, the inflammatory reaction may be enhanced by endotoxin release during the first 48 hours of treatment; however, definite progression at this stage is unusual and implies that either the organisms are resistant to therapy, or the patient is not instilling the drops as prescribed.^1^ If the patient is judged to be improving, the dose of antibiotics and/or antifungal drops should be reduced from hourly, to two-hourly, then four-hourly over the next two weeks in case of bacterial ulcers. For fungal ulcers, treatment should be continued with three-hourly drops for at least three weeks.

### Guidelines for referral to a tertiary centre

Immediate referral on **presentation** if:

- the ulcer is in an only eye
- the patient is a child
- there is impending or actual perforation
- a fungal corneal ulcer is suspected but KOH or other fungal stains are not available.

**Following initial treatment:** if cases of bacterial ulcer fail to show any improvement within three days, and fungal ulcers within a week, patients should be referred to a tertiary care centre.

**Figure:**
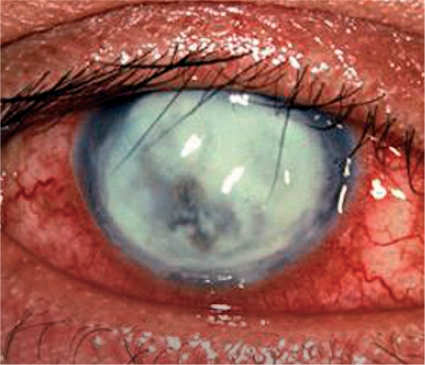
Figure 3. Subtotal fungal ulcer

Dr Whitcher/UCSF

## Management of corneal ulcer at tertiary level

Many tertiary eye care centers have their own protocol for the management of corneal ulcer. The management suggested is based on a WHO recommendation for suitable modification according to circumstances.^2^

### History, examination, and recording of findings

By the time patients have reached a tertiary centre, they will have travelled from one place to another with attendant hassles, received several treatments, may have lost faith in eye care personnel, and may already have run out of money, particularly in low-income countries. Attending to this situation is critically important in the overall care of corneal ulcer patients.

A careful history of the development of the disease may point to the existence of an underlying predisposing condition such as diabetes mellitus, immunosuppression due to local or systemic steroids (or other immunosuppressants), dacryocystitis, or other ocular conditions. A full list of drugs used by the patient should be obtained to ensure that drugs which have not helped in the past are not repeated; this may also help to discover possible drug allergies. Findings should be carefully noted on a standard form.

A meticulous corneal scraping subjected to laboratory processing often provides a sound guideline to treatment. See page 42.

### Hospitalisation

This provides patients with rest and adequate medication; they can also receive frequent follow-up, management of systemic problems such as diabetes, and further surgical intervention, if warranted.

### Treatment

The initial treatment depends on the results of the corneal scrape and the local pattern of pathogens and antibiotic resistance.

- If microscopy is negative, or it is not possible to perform a corneal scrape, or Gram-positive or Gram-negative bacteria are visualised, treat the patient with antibiotic eyedrops. Use either a combination of cefazolin 5% and gentamycin 1.4%, or fluoroquinolone monotherapy (eg. ciprfloxacin 0.3% or ofloxacin 0.3%). Drops should be given hourly to begin with for two days and then tapered, based on response.
- If microscopy reveals fungal hyphae, topical natamycin 5%, econazole 1% or amphotericin-B 0.15% should be used hourly for a week and then tapered.
- If the ulcer seems to respond well to treatment, continue therapy as before for two weeks for a bacterial ulcer and three weeks or more for fungal ulcer.
- If the response is poor and the culture shows growth of an organism, the choice of antibiotic is guided by the sensitivity reports.

Although a large number of antifungal drugs are available for systemic mycoses, only few are effective for treatment of corneal ulcers. The commonly recommended drugs are given in Table 2.

**Table 1:** Preparation of fortified antibiotic eye drops

Antibiotic	Method	Final concentration
Cefazolin/cefuroxime	Add 10 ml sterile water to 500 mg cefazolin powder; mix and use as topical drops. Shelf life: 5 days	50 mg/ml (5%)
Gentamicin (tobramycin)	Add 2 ml parenteral gentamicin (40 mg/ml) to a 5 ml bottle of commercial ophthalmic gentamicin (3 mg/ml)	14 mg/ml (1.4%)
Penicillin G	Add 10 ml of artificial tears to a 1 million unit vial of Penicillin G powder; mix, remove, and place into empty artificial tear bottle or xylocaine vials (30 ml)	100,000 units/ml
Vancomycin	Add 10 ml sterile water to a 500 mg vial of vancomycin powder; mix, add sterile cap, and use	50 mg/ml (5%)
Amikacin	Add 2 ml of parenteral amikacin containing 200 mg to 8 ml artificial tears or sterile water in a sterile empty vial.	20 mg/ml (2%)

**Table 2:** Commonly recommended antifungal drugs

Drug	Topical	Systemic
Amphotericin-B	0.15-0.5% drops	IV infusion
Natamycin	5% drops	Not available
Econazole	2% drops	Not available
Voricanozole	1% drops	Oral tablets 100–200 mg/day
Ketoconazole	2% drops	Oral tablets 200–600 mg/day
Miconazole	1-2% drops	Intravenous injection
Clotrimazole	1-2% ointment	Not available
Fluconazole	0.2-0.3% drops	Oral tablets 200 mg/day

However, except for natamycin and fluconazole, others are not available commercially for topical ocular use. The other antifungals have to be prepared using commercially available injectable forms such as amphotericin-B, miconazole, or raw materials such as clotrimazole and voriconazole.

Other agents such as polyhexamethylene biguanide (PHMB) 0.02%, chlorhexidine 0.02%, povidone iodine 1.5 to 5% and silver sulfadiazine 1% have been reported to possess variable antifungal activity and may be used if other drugs are not available.

Natamycin 5% suspension is recommended for treatment of most cases of filamentous fungal keratitis, particularly those caused by *Fusarium* sp. Topical miconazole 1% (not commercially available for topical use) can be used as adjunct or supportive therapy. Most clinical and experimental evidence suggests that topical amphotericin-B (0.15 to 0.5%) is the most efficacious agent available to treat yeast keratitis. Amphotericin-B is also recommended for fungal keratitis caused by *Aspergillus sp.* Oral ketaconazole (200–600 mg/day) may be considered as an adjunctive therapy in more severe fungal keratitis due to filamentous fungus. Oral fluconazole (200–400 mg per day) has been used successfully for severe keratitis caused by yeasts. Oral itraconazole (200 mg/day) has broad-spectrum activity against all *Aspergillus sp.* and *Candida* but has variable activity against *Fusarium* sp.

Fungal infection of the deep corneal stroma may not respond to topical antifungal therapy because of poor penetration of these agents in the presence of an intact epithelium. It has been reported that a 5 mm epithelial debridement (as a diagnostic scraping or therapeutic procedure) greatly enhances the penetration of antifungal drugs.

Animal experiments indicate that frequent topical application (every five minutes) for an hour can readily achieve therapeutic level.

## Surgical management

The range of surgical interventions available for management of corneal ulcer may include debridement, corneal biopsy, tissue adhesives, conjunctival flap, tarsorraphy, or therapeutic corneal graft. Evisceration of the eye is performed for severe pain, panophthalmitis, or life-threatening complications.

### Tarsorrhaphy

This is an old surgical technique that is still very useful today. In suppurative keratitis due to fungal and bacterial infections, tarsorrhapy is effective in promoting healing, provided the ulcer has been sterilised by effective antibacterial and/or antifungal treatment. Following central tarsorrhaphy, it can be difficult to instil drops and to see the cornea, so it is vital to ensure that the infection is under control before closing the eyelids. However, tarsorrhaphy often leads to rapid resolution of persistent epithelial defects, whatever the underlying cause. Once tarsorrhaphy is performed it is left in place for at least one to three months. There are different surgical techniques which are described well in many standard ophthalmic text books; however, simply suturing the lids together with a non-absorbable stitch can be effective.

### Conjunctival flap

The principle of this technique is to promote healing of a corneal lesion by providing adequate nutrition through the conjunctival blood vessels. The flap could be three types:

1. A total flap covering the entire cornea, called Gunderson's flap.
2. A pedicle (racquet) flap. A pedicle flap carries its own blood supply from the limbus and is useful for ulcers near the limbus.
3. A bucket handle flap. This carries its blood supply from both ends of the flap and may be less likely to retract. It is more useful for central corneal ulcers.

This procedure can be performed under local anaesthesia. Harvesting adequate bulbar conjunctiva in eyes which have had previous surgery may be difficult. The flap should be as thin as possible, with minimal adherent subconjunctival tissue. Following removal of any remaining corneal epithelium, the flap should be sutured to the cornea with 10-0 nylon sutures.

The conjunctival flap promotes healing by vascularisation. It is particularly useful in patients with impending perforation, when it may preserve the globe and allow subsequent corneal grafting. However, a flap may limit the penetration of topical antibiotics, so it should only be performed once the ulcer has been sterilised and the infection brought under control.

## Conclusion

Management of suppurative keratitis remains a major challenge worldwide, more so in low- and middle-income countries with inadequate health care resources. Although the outcome of treatment has improved significantly, many patients continue to deteriorate in spite of the best treatment that can be offered. The continued emergence of strains of microorganisms that are resistant to an ever-expanding range of antimicrobials poses an additional challenge. Further research related to prevention of suppurative keratitis and enhancing host resistance are two worthwhile goals to pursue. Large-scale public education programmes to sensitise those at risk of suppurative keratitis, and to encourage earlier presentation, should be undertaken. Coupled with this, education of practioners, general physicians, and other health workers, as well as general ophthalmologists, will go a long way towards ensuring correct diagnosis, appropriate treatment, and timely referral before extensive damage to the cornea occurs. Management of corneal abrasions at primary care levels within 48 hours has been demonstrated by various studies to be the best way to prevent corneal ulcers in low- and middle-income countries.^3^–^6^ This could be adopted in any population and is cost effective both for health providers and the patient.
